# Acupuncture Therapy on Patients with Flaccid Hemiplegia after Stroke: A Systematic Review and Meta-Analysis

**DOI:** 10.1155/2022/2736703

**Published:** 2022-01-10

**Authors:** Yang Tu, Wei Peng, Jun Wang, QingHong Hao, Yang Wang, Hui Li, TianMin Zhu

**Affiliations:** ^1^School of Health Preservation and Rehabilitation, Chengdu University of Traditional Chinese Medicine, Chengdu 610075, China; ^2^School of Acupuncture and Tuina, Chengdu University of Traditional Chinese Medicine, Chengdu 610075, China; ^3^School of Preclinical Medicine, Chengdu University, Chengdu 610106, China

## Abstract

**Background:**

Acupuncture is a commonly used complementary treatment for flaccid hemiplegia caused by stroke, but evidences from previous randomized trials were inconclusive. The purpose of this study was to evaluate the efficacy and safety of acupuncture in a comprehensive synthesis.

**Methods:**

We searched literature from eight databases from their inception to December 2020. We included randomized controlled trials of acupuncture for the treatment of flaccid hemiplegia following stroke. The meta-analysis was carried out using Review Manager 5.3 and Stata 16.0. The main indicator was the Fugl-Meyer Assessment scale. The modified Barthel Index scale, Quality Of Life Assessment scale, Mini-Mental State Examination scale, Berg Balance Scale, Neurological Deficit Assessment scale, and the treatment effective rate were used to measure the secondary indicators. Adverse events from individual studies were used to determine safety.

**Results:**

Our search returned 7624 records, of which 27 studies involving a total of 1,293 patients fulfilled our inclusion criteria. To be noted, our results indicated that significant improvements in the scores of the primary indicator showed better clinical scores among the three groups with acupuncture than without acupuncture: acupuncture compared with rehabilitation, 13.53 (95% CI 11.65–14.41, *P* < 0.01); acupuncture plus rehabilitation compared with rehabilitation, 9.84 (95% CI 6.45–13.24, *P* < 0.01, *I*^2^ = 98%); and acupuncture plus Western medicine therapy compared with Western medicine, 16.86 (95% CI 15.89–17.84, *P* < 0.01, *I*^2^ = 38%), and the secondary indicators showed the same tendency.

**Conclusion:**

Acupuncture was effective and safe in the patients with flaccid hemiplegia after stroke, although there was high heterogeneity between studies.

## 1. Strengths and Limitations of This Study

1. This systematic review offered a thorough assessment of the efficacy and safety of acupuncture treatment for individuals with flaccid hemiplegia following a stroke. Compared with the nonacupuncture group, the scores of the acupuncture group were significantly improved in the following aspects: motor function, ability of daily living, cognitive function, quality of daily life, neurological deficit, and the clinical effective rate, indicating that acupuncture was effective and short-term safe for patients with poststroke flaccid hemiplegia.
2. The comprehensiveness and methodological quality of the major studies included in this evaluation have a substantial impact on the results' trustworthiness.
3. This systematic review's protocol has been registered in PROSPERO, and it has been conducted and reported in complete compliance with PRISMA guidelines. However, selection bias may exist as we only searched the English and Chinese databases.

## 2. Background

Stroke is regarded as the leading cause of death and disability worldwide. [1] Hemiplegia after stroke is one of the primary functional disorders in patients with stroke. Paresis and spastic phases are common in hemiplegia induced by stroke. Most of the limbs show flaccid hemiplegia in the early stages following a stroke. The upper neuron of the patient loses control of the lower center, and the spinal cord center that has not been physically injured has shock, resulting in flaccid hemiplegia, namely Brunnstrom I∼II. The natural healing duration in the flaccid hemiplegia stage is roughly two weeks. Reconstruction of motor function is crucial for the social reintegration of stroke patients. However, the longer the duration of hemiplegia, the worse the prognosis, and the higher the residual physical disability rate [2]. After stroke by three months, there is still considerable room for improvement in all measures: 85% of persons were even impaired on gait speed, 78% had not reached age-specific norms for upper extremity function, 68% even demonstrated delayed physical mobility, 37% required some assistance with necessary activities of daily living, and 29% were even impaired on balance [3]. Accordingly, the primary objective of poststroke therapy is to shorten the duration of flaccid hemiplegia.

At present, the main treatments for poststroke flaccid hemiplegia include exercise rehabilitation training (e.g., continuous passive motion), noninvasive brain stimulation (e.g., transcranial direct current stimulation (tDCS) and transcranial magnetic stimulation (rTMS)), and physical therapy modalities (e.g., neuromuscular electrical stimulation (NMES)). However, there are certain disadvantages to these treatments. In terms of unpleasant symptoms, continuous passive motion is preferable to a therapist-directed range of motion exercise (especially shoulder stability). While the passive range of motion may help with brain healing, its capacity to cause significant motor alterations is debatable [4–6]. The ideal dose and montage, long-term safety in stroke patients, and the impact magnitude of tDCS are all challenging issues in tDCS research for stroke rehabilitation. The US Food and Drug Administration (FDA) licensed repetitive transcranial magnetic stimulation for “drug-resistant depression,” [7] but its clinical utility in stroke recovery is yet unknown [8,9]. Electrical stimulation of the peripheral neuromuscular system has not had beneficial outcomes in studies [10]. More importantly, there were inadequate robust data to inform its therapeutic usage for neuromuscular retraining following stroke, according to a Cochrane review published in 2006 [11]. Questions remain unanswered regarding the type, dose, and timing of these peripheral stimulation techniques.

Acupuncture has been practiced clinically in China for more than 3000 years and has been widely used to treat various diseases, especially in stroke rehabilitation treatment [12,13]. Acupuncture is internationally recognized as an essential treatment for multiple conditions associated with stroke. Besides, acupuncture is recommended by the World Health Organization (WHO) as an alternative and complementary strategy for stroke treatment and for improving stroke care [14]. Its advantages in the treatment of stroke have recently been highlighted in research [15–17]. Guo [18] believed that acupuncture was effective in the rehabilitation of stroke, but Zhu [19] found that the addition of acupuncture to a regimen of conventional physical therapy did not result in further improvement in motor function. Taking these inconsistent findings into account, a systematic evaluation is needed to summarize them to reach a consistent conclusion.

Despite the numerous clinical researches on acupuncture, many professionals continue to have reservations regarding its efficacy and safety. Hence, the goal of this systematic review and meta-analysis is to determine the effectiveness and safety of acupuncture treatment for flaccid hemiplegia following stroke.

## 3. Methods

The protocol of this systematic review was developed and submitted to PROSPERO, and the registration number is CRD42020180411.

### 3.1. Data Sources and Search Strategy

A systematic search was conducted in the following databases: China Biology Medicine (CBM), China National Knowledge Infrastructure (CNKI), Wan Fang Data, the Chinese Science and Technology Periodical Database (VIP), PubMed, EMBASE, the Cochrane Library, and Web of Science from the inception to December 2020. All searches were performed by a biomedical information specialist of the medical library, with an exhaustive set of search terms related to “acupuncture,” “stroke,” “randomized controlled trial,” and “flaccid hemiplegia.”

### 3.2. Inclusion and Exclusion Criteria

Studies were included if they fulfilled the following criteria: (1) randomized controlled trials (RCTs); (2) patients were diagnosed with flaccid paralysis after stroke, regardless of the type of stroke (hemorrhagic stroke or ischemic stroke), the diseased brain area, the affected limb, gender, age, ethnicity, and nationality; and (3) acupuncture used as an intervention (such as electroacupuncture, needle warming therapy, scalp acupuncture, hand-foot acupuncture, tongue acupuncture, auricular acupuncture, and nerve trunk stimulation therapy), regardless of the number and duration of the treatment. Studies were excluded if they did not conform to the study type (opinions, case reports, case series, conference papers, editorials, abstracts, and crossover studies) and had inadequate information.

### 3.3. Outcome Assessment

The primary outcome measure was assessed by the Fugl–Meyer Assessment (FMA) scale, which was usually used to assess motor function. The secondary outcomes included modified Barthel Index (MBI) scale, Quality of Life (QOL) Assessment scale, Mini-Mental State Examination (MMSE) scale, Berg Balance Scale (BBS), Neurological Deficit (ND) Assessment scale, or other scale data related to flaccid hemiplegia. Safety was evaluated by any recorded adverse events reported by individual study.

### 3.4. Data Extraction and Collection of Adverse Events

**Figure 1:**
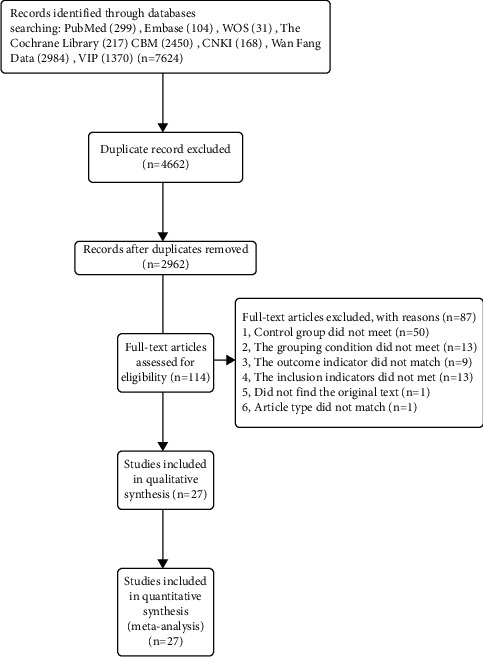
Flow chart of trial selection process.

Two independent reviewers (YT and JW) independently screened the literatures according to the predefined inclusion criteria based on the title and abstract. The two examiners then separately reviewed the full text based on the retrieved results. The included studies were cross-checked by two reviewers. Disagreements were resolved through discussion or consensus with third reviewers (WP). The research selection process is shown in the PRISMA flow chart in Figure 1.

### 3.5. Quality Assessment

**Figure 2:**
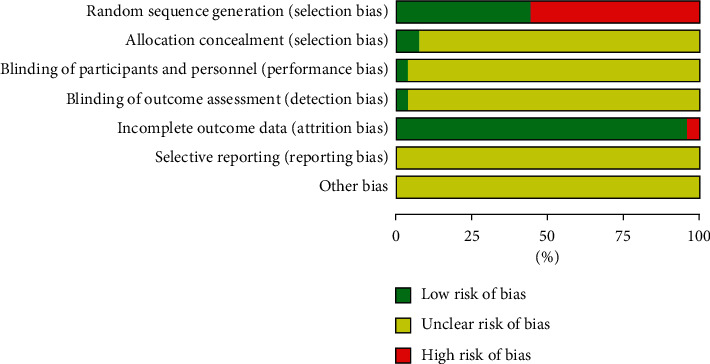
Risk of bias in the involved studies, assessed by using the Cochrane Collaboration's risk-of-bias tool: high risk of bias (+); unclear risk of bias (?); and low risk of bias (−).

**Figure 3:**
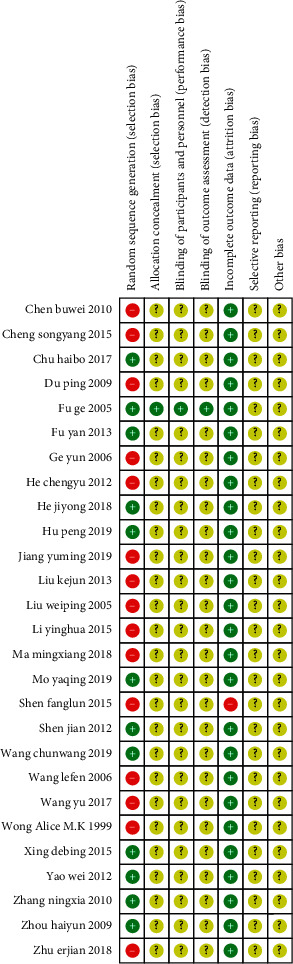
Graph of the risk of bias: percentage of all studies included.

The two authors conducted an independent assessment of the risk of bias using the Cochrane risk-of-bias tool to assess the methodological quality of the included studies. There were seven items in total, and each item was determined to be one of the following: “low risk of bias,” “unclear risk of bias,” and “high risk of bias.” If all seven items were assessed as having a low risk of bias, the study was rated as high quality. If one or more items were assessed as having a high or unclear risk of bias, the study was rated as low quality (see Figures 2 and 3).

### 3.6. Data Analysis

All statistical analyses were performed using Review Manager 5.3 and Stata 16.0. The confidence interval for all data was 95% (95% CI). Continuous outcomes were evaluated using mean difference (MD) or STD mean difference (SMD), while the dichotomous outcomes were evaluated by risk ratio (RR) or odds ratio (OR). Statistical heterogeneity was assessed by *I*^2^ statistics and chi-square test *P* value. If the chi-square test *P* value was greater than 0.05 or the *I*^2^ statistic was less than 50%, the combination of RR or OR for each study using the fixed-effects model was applied to the dichotomous data, and the combination of MD or SMD for each study using the fixed-effects model was used for continuous data. Otherwise, a random-effects model was used to assess the effects of the intervention more conservatively. Sensitivity analysis and subgroup analysis were employed to further explore the source of heterogeneity. Egger's test was used to assess publication bias.

## 4. Results

### 4.1. Study Characteristics

A total of 7624 records were obtained from the nine databases. After removing the duplicates, 2962 articles were left. A total of 2848 articles were then excluded because they did not meet the inclusion criteria. As a result, 27 randomized controlled trials [20–46] were included in the qualitative analysis (see Figure 1). Study characteristics of the included literatures are summarized and listed in Table 1.

### 4.2. Description of Participants

The review and meta-analysis included 27 studies involved 1,293 patients, of which 648 were in the trial group (381 men and 267 women) and 635 were in the control group (372 men and 263 women). All studies were conducted and published between 1999 and 2019. Regarding the sample size, the trials included a maximum of 146 people and a minimum of 40 people.

### 4.3. Description of Interventions

For intervention, one study adopted acupuncture, sixteen studies adopted acupuncture combined with rehabilitation, four studies adopted electroacupuncture combined with rehabilitation, one study adopted scalp acupuncture combined with rehabilitation, one study adopted electroacupuncture, one study adopted electroacupuncture combined with Western medicine, and three studies adopted acupuncture combined with Western medicine. The detailed intervention characteristics of the included literatures are summarized and listed in Table 1.

### 4.4. Description of Outcomes

In all the involved studies, seventeen studies used FMA scale to assess limb function, and eighteen studies used MBI scale to assess daily living ability. Eight studies use ND scale to assess neurological deficit. One study used MAS scale to assess spasticity. To evaluate the quality of daily life, three studies adopted the QOL scale, while two studies adopted BBS to value the balance function.

### 4.5. The Efficiency of Acupuncture Therapy

#### 4.5.1. Acupuncture Compared with No Treatment

**Figure 4:**
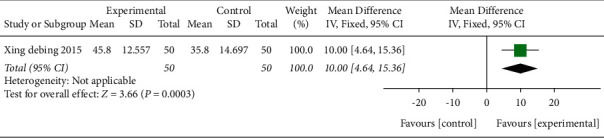
Forest plot (acupuncture compared with no treatment).

One study [43] including 40 patients adopted MBI scale as the main outcome index in Figure 4. After combining and analyzing the data, the results showed that acupuncture improved the daily living ability (MD 10.00, 95% CI 4.64–15.36, *P* < 0.01).

#### 4.5.2. Acupuncture Compared with Rehabilitation

**Figure 5:**
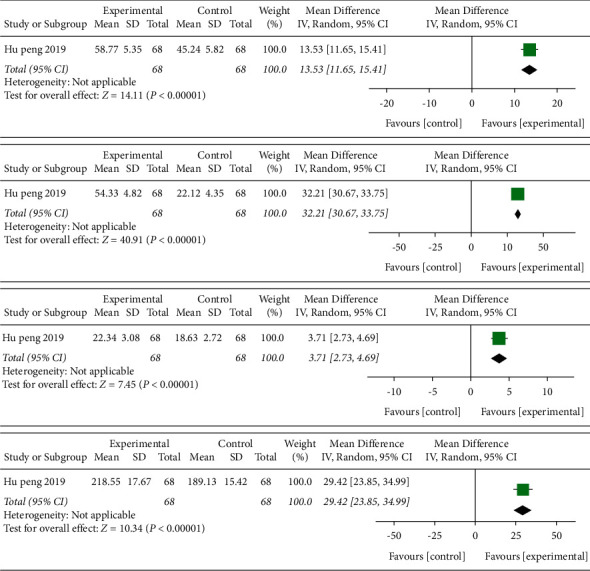
Forest plot (acupuncture compared with rehabilitation).

One study [33] containing 68 cases compared efficacy of acupuncture with rehabilitation in Figure 5. The MD value of FMA, MBI, MMSE, and QOL was 13.53 (95% CI 11.65–14.41, *P* < 0.01); 32.21 (95% CI 30.67–33.75, *P* < 0.01); 3.71 (95% CI 2.73–4.69, *P* < 0.01); and 29.42 (95% CI 23.85–34.99, *P* < 0.01), respectively.

#### 4.5.3. Acupuncture plus Rehabilitation Compared with Rehabilitation

Twenty-one studies [22,28,30,32,34,39,41, 42,44–46] reported acupuncture plus rehabilitation compared with rehabilitation, including 1845 cases.

**Figure 6:**
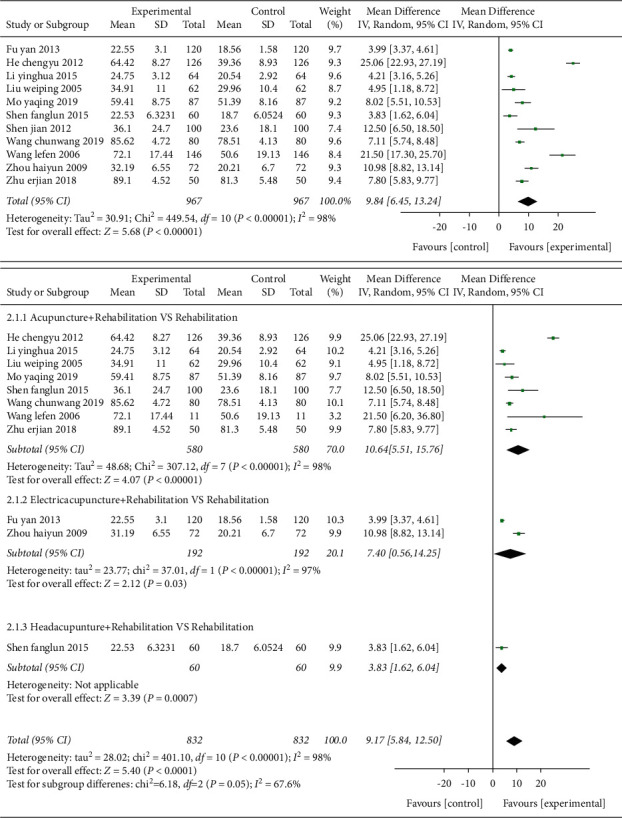
Forest plot (acupuncture plus rehabilitation compared with rehabilitation and its subgroup analysis on FMA).

*(1) FMA and Subgroup Analysis of Interventions for FMA.* Eleven studies [22,23,26,27,31,36,38,39,44–46] used FMA scale as an outcome indicator in Figure 6, with MD value of 9.84 (95% CI 6.45–13.24, *P* < 0.01, *I*^2^ = 98%).

Subgroup analysis showed that the rehabilitation combined with acupuncture regardless of its subtypes ameliorated more than the rehabilitation treatment alone in the scores on the motor function of the limbs assessed by FMA scale. After combining effect size, the MD value was 9.17 (95% CI 5.84–12.5, *P* < 0.01, *I*^2^ = 98%). Eight studies [22,23,27,31,38,39,45,46] compared acupuncture plus rehabilitation with rehabilitation, and the MD value was 10.64 (95% CI 5.51–15.76, *P* < 0.01, *I*^2^ = 98%); two studies [26,44] were electroacupuncture plus rehabilitation therapy compared with rehabilitation, and the MD value was 7.4 (95% CI: 0.56–14.25, *P*=0.03, *I*^2^ = 97%); and one study [23] was scalp acupuncture combined with rehabilitation therapy compared with rehabilitation therapy, and the MD value was 3.83 (95% CI 1.62–6.04, *P* < 0.01).

**Figure 7:**
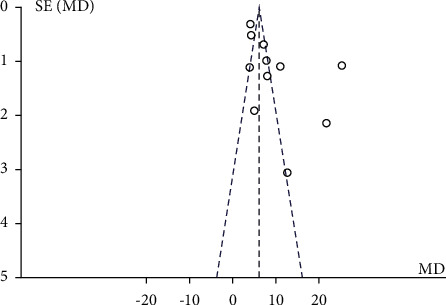
Funnel plot (acupuncture plus rehabilitation compared with rehabilitation on FMA).

After subgroup analyzing and combining data, substantial heterogeneity was observed (*I*^2^ = 67.6%, *P*=0.05). Through sensitivity analysis, no obvious source of heterogeneity was found. After meta-regression analysis, it was found that the sample size was a source of heterogeneity. Egger's test indicated that there was no obvious publication bias (*P*=0.058) in Figure 7.

**Figure 8:**
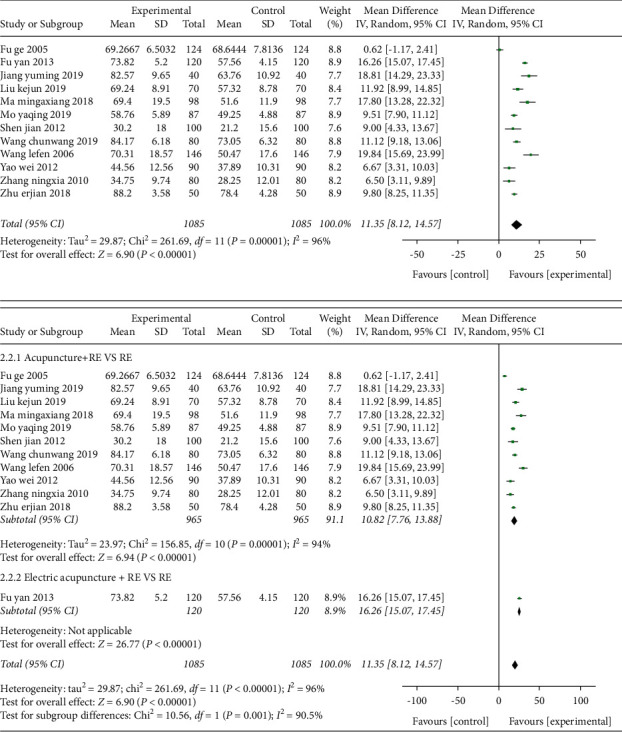
Forest plot (acupuncture plus rehabilitation compared with rehabilitation and its subgroup analysis on MBI).

*(2) MBI and Subgroup Analysis of Interventions for MBI.* Twelve studies [22,24,28,30,32,35,36,38,39,41,44,45] used MBI as the outcome indicator, involving a total of 1085 cases in Figure 8. The MD value was 11.35 (95% CI 8.12–14.57, *P* < 0.01, *I*^2^ = 96%).

**Figure 9:**
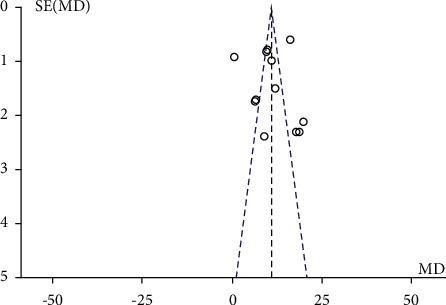
Funnel plot (acupuncture plus rehabilitation compared with rehabilitation on MBI).

Subgroup analysis showed that the acupuncture combined rehabilitation group and the electroacupuncture combined rehabilitation treatment group were superior to the simple rehabilitation treatment group in improving the scores of the ability of daily living. Eleven studies [22,24,28,30,32,35,36,38,39,41,45] were acupuncture combined with rehabilitation treatment group compared with rehabilitation treatment group, and the MD value was 10.82 (95% CI 7.76–13.88, *P* < 0.01, *I*^2^ = 94%). One study [44] adopted electroacupuncture combined with rehabilitation therapy compared with rehabilitation treatment group, and the MD value was 16.26 (95% CI 15.07–17.45, *P* < 0.01, *I*^2^ = 90.5%). After sensitivity analysis, two studies [24,44] were the main sources of heterogeneity. The heterogeneity was slightly reduced after the removal of those studies (*I*^2^ = 84%, *P* < 0.01). Meta-regression analysis of publication year, sample size, and intervention methods were not associated with heterogeneity. Egger's test indicated that there was no obvious publication bias (*P*=0.964) in Figure 9.

**Figure 10:**
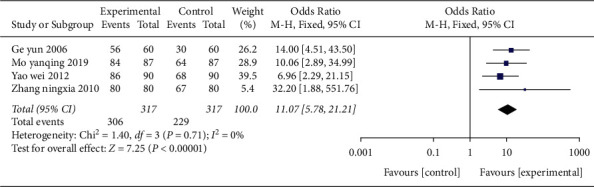
Forest plot (acupuncture plus rehabilitation compared with rehabilitation on the effective rate).

*(3) The Effective Rate.* Four studies [25,35,41,45] adopted the effective rate as the outcome indicator in Figure 10, including a total of 317 cases. The OR value was 11.07 (95% CI 5.78–21.21, *P* < 0.01, *I*^2^ = 0%). It indicated that the effective rate of acupuncture combined with rehabilitation group was higher than that of the simple rehabilitation group.

**Figure 11:**
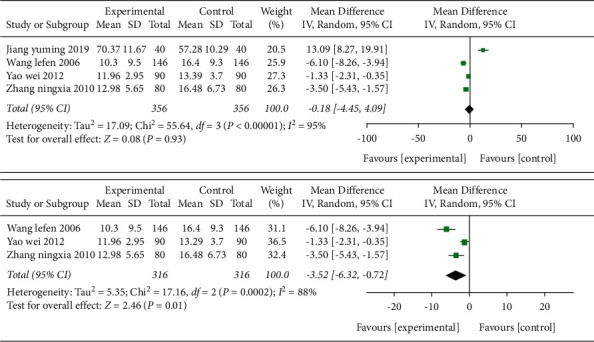
Forest plot (acupuncture plus rehabilitation compared with rehabilitation on ND).

*(4) ND.* Four studies [28,35,39,41] adopted ND as the outcome indicator in Figure 11, and a total of 356 cases were included. After combining the effect size, the results showed that the MD value was -0.18 (95% CI −4.45 to 4.09, *P*=0.93, *I*^2^ = 95%).

After sensitivity analysis, one study [28] was the source of heterogeneity. After elimination, the combined effect size showed that acupuncture combined with rehabilitation therapy was superior to the rehabilitation therapy group in terms of improving the scores on neurological function, and the MD value was -3.52 (95% CI −6.32 to −0.72, *P*=0.01, *I*^2^ = 88%).

#### 4.5.4. Acupuncture plus Western Medicine Therapy Compared with Western Medicine Therapy

Four studies [20,21,29,40] reported acupuncture plus Western medicine therapy compared with Western medicine therapy, involving a total of 438 cases. In terms of different intervention groups and control groups, one study [20] compared electroacupuncture plus Western medicine treatment with Western medicine treatment alone, and three studies [21, 29, 40] compared acupuncture plus Western medicine treatment with Western medicine treatment alone. In response to different outcomes, we made the following analysis.

**Figure 12:**
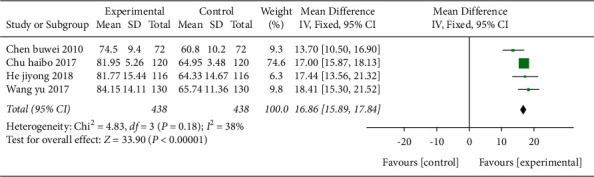
Forest plot (acupuncture plus Western medicine therapy compared with Western medicine therapy on FMA).

*(1) FMA*. Four studies [20,21,29,40] used FMA as the outcome indicator in Figure 12, and a total of 438 cases were included. The results showed that acupuncture combined with Western medicine was better than Western medicine alone in improving the motor function of the patient's limbs with MD value of 16.86 (95% CI 15.89–17.84, *P* < 0.01*I*^2^ = 38%).

**Figure 13:**
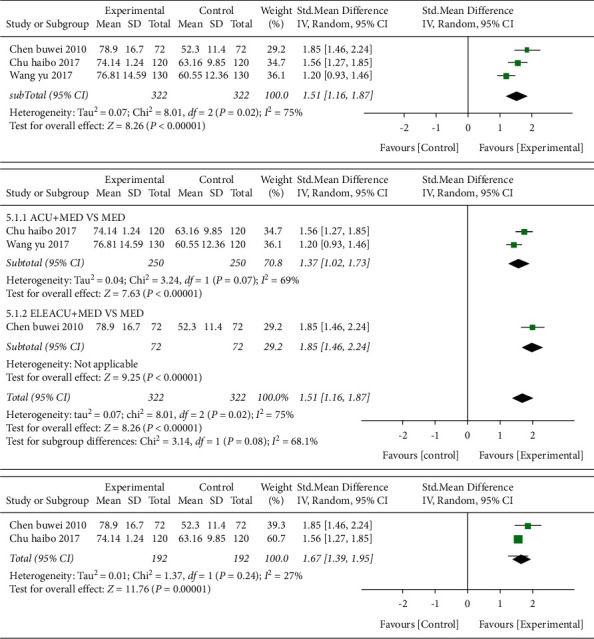
Forest plot (acupuncture plus Western medicine therapy compared with Western medicine therapy on MBI).

*(2) MBI and Subgroup Analysis of Interventions for MBI.* Three studies [20,21,40] used MBI as the outcome indicator and included a total of 322 cases in Figure 13. The results showed that the SMD value was 1.51 (95% CI 1.61–1.87, *P* < 0.01, *I*^2^ = 75%).

Two of them [21,40] were acupuncture combined with Western medicine treatment compared with Western medicine treatment, and the SMD value was 1.37 (95% CI 1.02–1.73, *P* < 0.01, *I*^2^ = 69%). One of them [20] was the combination of electroacupuncture and Western medicine treatment compared with Western medicine treatment alone. The SMD value was 1.85 (95% CI 1.46–2.24, *P* < 0.01).

After merging the subgroup analysis data, it suggested that there was still a high degree of heterogeneity (*P*=0.02, *I*^2^ = 75%). After sensitivity analysis, it was found that one study [40] was the main source of heterogeneity. The heterogeneity was significantly reduced after removal (*I*^2^ = 27%, *P* < 0.01).

**Figure 14:**
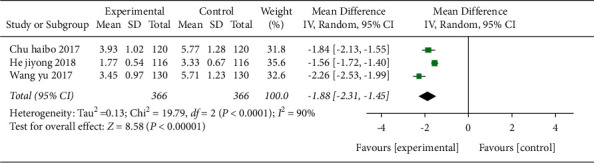
Forest plot (acupuncture plus Western medicine therapy compared with Western medicine therapy on ND).

*(3) ND and Subgroup Analysis of Interventions for ND.* Three studies [21,29,40] used ND as the outcome indicator, which included a total of 366 cases in Figure 14. Those studies compared acupuncture combined with Western medicine treatment compared with Western medicine treatment alone. The results showed that the MD value was -1.88 (95% CI −2.31 to −1.45, *P* < 0.01, *I*^2^ = 90%), indicating that acupuncture combined with Western medicine was superior to Western medicine alone in reducing the neurological deficit of patients.

After sensitivity analysis, it was found that Wang yu [40] may be the main source of heterogeneity, and the heterogeneity was slightly reduced after removal (*I*^2^ = 63%, *P*=0.1). Meta-regression analysis of publication year and sample size did not find obvious sources of heterogeneity.

**Figure 15:**
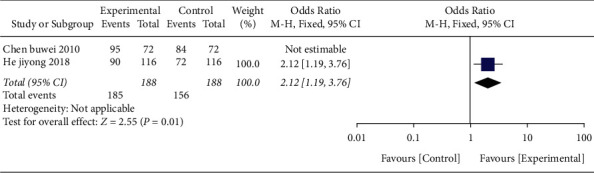
Forest plot (acupuncture plus Western medicine therapy compared with Western medicine therapy on the effective rate).

*(4) The Effective Rate.* Two studies [20,29] took clinical effectiveness as the outcome indicator in Figure 15 and included a total of 188 cases. After the data were merged, it was shown that one piece of research data [20] could not be evaluated. After the combined effect size, the OR value was 2.12 (95% CI 1.19–3.76, *P*=0.01), and the combination of acupuncture and Western medicine treatment was superior to Western medicine treatment alone in terms of effective numbers.

## 5. Discussion

### 5.1. Summary of the Results

In this study, we evaluated the efficacy and safety of acupuncture in the treatment of flaccid paralysis. Compared with the group without acupuncture intervention, the group with acupuncture intervention significantly improved the clinical scores, in terms of motor function, ability of daily living, cognitive function, quality of daily life, neurological deficit, and clinical effective rate, indicating that patients with flaccid hemiplegia could benefit from acupuncture.

This study adopted American Heart Association/American College of Cardiology Guidelines [47] to evaluate the effectiveness and safety of acupuncture in the treatment of flaccid hemiplegia after stroke. We searched eight Chinese and English databases from their inception to December 2020, and 27 RCTs were reviewed and involved in a meta-analysis. The patients with flaccid hemiplegia after stroke could benefit from acupuncture therapy in terms of motor function (FMA), ability of daily living (MBI), cognitive function (MMSE), quality of daily life (QOL), neurological deficit (ND), and the effective rate. The meta-analysis showed that patients in the acupuncture group had a better quality of life and functional recovery. After the methodological quality assessment, all studies were classified as low quality, and no adverse events were reported in the 27 studies, indicating that acupuncture was safe in the short term, but the long-term safety could not be assessed.

### 5.2. Comparison with and Description of Similar Studies

Cochrane's comments indicate that although acupuncture appears to be safe, there is no clear evidence that it was beneficial [48]. There was still no significant evidence to support the effectiveness of acupuncture treatment of flaccid hemiplegia after stroke in the previous studies. The results of this study provided a summary of the existing evidence regarding the efficacy and safety of acupuncture in patients with flaccid hemiplegia after stroke till December 2020. To the best of our knowledge, we expanded the scope of our search to find that this study is the first systematic review and meta-analysis to evaluate the efficacy and safety of acupuncture on the treatment of flaccid hemiplegia after stroke in the eight literature databases. We found that compared with participants who received only rehabilitation treatment, Western medicine therapy, or blank control, participants who received acupuncture treatment showed a significant improvement in the scores of scales, and the Fugl-Meyer Assessment scale as the main indicator showed that the emerged mean difference was divided into 13.53 (95% CI 11.65–14.41, *P* < 0.01), 9.84 (95% CI 6.45–13.24, *P* < 0.01, *I*^2^ = 98%), and 16.86 (95% CI 15.89–17.84, *P* < 0.01*I*^2^ = 38%). The results suggested that patients with flaccid hemiplegia after stroke could benefit from acupuncture. The secondary indicators also showed the same tendency.

Compared with three retrospective studies [49–51] of acupuncture therapy on spasticity after stroke that were similarly to flaccid paralysis, published in 2014, 2015, and 2017, respectively. The effect of acupuncture on patients with stroke observed in this study is consistent with the studies published in 2015 and 2017. The study [51] published in 2014 found that acupuncture had no effect on clinical outcome (the modified Ashworth scale, MAS) and physiological indicators (H reflex/M response). The differences in disease stage selection and study design might account for the differences in the effect of acupuncture. The study [50] published in 2015 used MAS as the main evaluation result, and the results showed that acupuncture could significantly reduce spasticity after stroke. The study [49] published in 2017 used MAS as the evaluation index to show that electroacupuncture reduced upper extremity spasm, and the FMA as the evaluation index showed that acupuncture improved overall motor function. At the same time, for lower extremity spasm, acupuncture also showed significant additional effects on lower extremity motor function and activities of daily living. There was no obvious additional effect on upper limb function.

In the past studies on spastic paralysis, most studies used the Fugl–Meyer Assessment scale and the Fugl-Meyer Assessment scale as subjective evaluation indicators, and some studies used H reflex/M response as objective physiological indicators. The studies involved in this study mostly use subjective scales to evaluate the effect of acupuncture, lacking objective physiological indicators. No matter in the stage of flaccid hemiplegia or spastic paralysis after stroke, most studies used scales related to limb motor function, ability of daily living, and neurological deficits. Due to the selection of partial scales as the outcome indicators, most studies did not use Manual Muscle Test scale combined with modified Ashworth scale to assess the condition of muscle strength and muscle tension, and lacked objective physiological indexes, resulting in imperfect efficacy assessments. The three studies all searched the Chinese and English literature databases, and the Korean-related literature databases were also searched in the study of 2014 and 2015. Although we have searched a large number of different databases, due to language limitations, the databases of countries that conduct more acupuncture trials such as South Korea and Japan had not been searched, which might cause some omissions in clinical trials.

### 5.3. Limitations of the Results

There were 3 limitations in this study. First, the included 27 studies had methodological flaws and were assessed as being of low quality. However, the reasons for its low quality should be further analyzed in detail. In most studies, the authors did not clearly state the random schemes, blind methods, allocation concealment, and other biases in the research process. Therefore, we could not judge whether the author of the article had not performed these steps, or whether they have been performed but had not been noted in the article. Researchers were unable to make specific judgments on the information due to irregular writing. Second, there was obvious heterogeneity in the scores of the Fugl-Meyer Assessment scale, the modified Barthel Index scale, and the Neurological Deficit Assessment scale. They were one of the main measurement indicators. In the subgroup analysis, the heterogeneity decreases with the intervention. At the same time, after excluding some studies through the sensitivity analysis, the heterogeneity was reduced. Meta-regression analysis found that sample size was one of the sources of heterogeneity. The diagnostic criteria of flaccid hemiplegia after stroke and the sample size were different, and acupuncture in a variety of forms, the selection of acupoints, acupuncture depth and frequency, amount of acupuncture stimulating, acupuncture operator and outcome indicators evaluation personnel qualification, and practice of the fixed number of years were different. Therefore, it might have a certain influence on the clinical efficacy and evaluation. Because the number of experimental data was not described in the study, the relevant experimental data could not be obtained after contacting the author, and the heterogeneity test of these items could not be completed. Therefore, these might be the source of heterogeneity. Third, the publication bias test was performed on groups of more than 10 studies, considering the existence of publication bias. The results of Egger's test of the Fugl-Meyer Assessment scale and the modified Barthel Index scale were *P*=0.058 and *P*=0.964, indicating that there was no obvious publication bias. Due to the limited number of trials, our findings should be treated with caution. In addition, all the evaluation indexes included in the studies were subjective judgment indexes such as the Fugl-Meyer Assessment scale and the modified Barthel Index scale, which were easily affected by the experience of clinicians and reviewers. Therefore, the existence of bias was easy to be detected.

Due to these limitations, the results of our study should be interpreted more cautiously. No adverse events were reported in the included 27 studies. Acupuncture treatment did not seem to cause serious adverse events and seemed to be safe in the short term, but its long-term safety was unknown, and we could not find enough evidence to support this view.

### 5.4. Suggestions for Future Studies

Nowadays, acupuncture has been widely used to treat hemiplegia caused by stroke. When patients with stroke are in a state of hemiplegia, acupuncture can promote the rehabilitation of the patient's muscle strength and muscle tension, and improve the motor function of the limbs, which can effectively prevent the occurrence of various complications [52–57]. However, the current research on acupuncture treatment of stroke is very abundant, and the research on acupuncture's mechanism to improve the motor function of the limbs mainly focuses on the spasticity and recovery period. There are insufficient studies on acupuncture treatment of the flaccid stage after stroke.

According to the available evidence, acupuncture treatment was beneficial to the recovery of patients with flaccid hemiplegia after stroke, although most of the included studies had methodological flaws. It was not yet possible to determine overall that acupuncture was better than other therapies in the treatment of flaccid hemiplegia after stroke. It is recommended that future study should be based on the CONSORT statement and the STRICTA consensus reporting plan and report the details of acupuncture treatment, such as the number of needles used in each treatment unit, specific acupuncture stimulation methods, needle insertion depth, needle response, treatment course, the qualifications of acupuncturist, evaluator, and the number of years of clinical practice, providing a standard and clear treatment plan, which will help to develop a more effective treatment prescription and evaluation system. In addition, it is necessary to expand the sample size, carry out multicenter, high-quality randomized controlled trials to confirm the conclusions, increase observation and follow-up to further clarify the long-term efficacy and safety of acupuncture treatment, and apply acupuncture to patients with flaccid hemiplegia after stroke treatment provides a more reliable clinical basis.

## 6. Conclusion

The findings in this study suggested that acupuncture as a complementary therapy was effective and short-term safe for patients with poststroke flaccid hemiplegia. However, the methodological deficiencies in previous studies have led to the call for carefully designed larger studies to confirm the potential benefit of acupuncture for patients with poststroke flaccid hemiplegia rehabilitation in future.

## Figures and Tables

**Table 1:** Characteristics and details of interventions of included studies.

Study	Sample size	Age		Duration (day)		Criteria for grading		Criteria for grading flaccid hemiplegia	Intervention		Frequency and retention time	Length of treatment	Outcomes
		Experimental	Control	Experimental	Control	Chinese medicine	Western medicine		Experimental	Control			
Chen 2010 [20]	72	54.4	55.4	Not described	Not described	Not described	Not described	Not described	Electroacupuncture + Western medicine	Western medicine	Once/day, not described	28 d	ND, FMA, MBI
Songyang 2015 [37]	120	65	64	4.5	4.7	Not described	Not described	Not described	Acupuncture + rehabilitation	Rehabilitation	5 times/week, 30 min	30 d	MBI
Chu et al. 2017 [21]	120	71 ± 8	69 ± 8	8.2 ± 3.7	9.2 ± 3.3	Not described	E	Not described	Acupuncture + Western medicine	Western medicine	6 times/week, 30 min	2 W	FMA, ND, MBI
Ping and Chen 2009 [34]	78	60.5 ± 15.4	63.4 ± 14.9	Not described	Not described	Not described	C	Not described	Electroacupuncture + rehabilitation	Rehabilitation	Not described, 30 min	30 d	MAS
Ge 2005 [24]	124	62.15 ± 8.53	65.76 ± 9.19	Not described	Not described	Not described	C	Not described	Acupuncture + rehabilitation	Rehabilitation	5 times/week, 30 min	4 W	FMA, QOL
Yan 2013 [44]	120	63.07 ± 5.12	62.05 ± 6.25	16.65 ± 2.25	13.25 ± 5.25	Not described	D	Not described	Electroacupuncture + rehabilitation	Rehabilitation	Once/day, 30 min	4 W	FMA, MBI, BBS
Ge Yun and Zhu 2006 [25]	60	62.4	68.8	3.8	4.6	B	C	Not described	Acupuncture + rehabilitation	Rehabilitation	5 times/week, 30–45 min	8 W	ND
Chengyu 2012 [27]	126	60 ± 6.9	59 ± 7.6	9 ± 1.8	9 ± 2.1	I	Not described	Not described	Acupuncture + rehabilitation	Rehabilitation	Once/day, 30 min	6 M	FMA
Jiyong 2018 [29]	116	69 ± 6	67 ± 7	7.3 ± 3.5	7.1 ± 3.3	I	J	Not described	Acupuncture + Western medicine	Western medicine	6 times/week, 30 min	14 d	FMA, ND
Peng et al. 2019 [33]	68	62 ± 10	62 ± 8	31 ± 8.4	29.7 ± 9.2	B	D	K	Acupuncture	Rehabilitation	6 times/week, 6h	4 W	FMA, MBI, MMSE, QOL
Yuming and Xi 2019 [28]	40	Not described	Not described	Not described	Not described	Not described	G	Not described	Acupuncture + rehabilitation	Rehabilitation	Once/day, 20 min	4 W	ND, MBI, BBS
Yinhua 2015 [46]	64	67.38 ± 7.16	67.21 ±	Not described	Not described	A	E	Not described	Acupuncture + rehabilitation	Rehabilitation	Not described, 30 min	Not described	FMA
Kejun 2013 [30]	70	64.7 ± 8.6	64.5 ± 8.3	4.5 ± 2.2	4.7 ± 2.1	I	Not described	Not described	Acupuncture + rehabilitation	Rehabilitation	Once/day, 30 min	30 d	MBI
Liu et al. 2005 [31]	62	61.46 ± 9.23	60.89 ± 10	5.727 ± 2.1	5.697 ± 2.3	Not described	C	Not described	Acupuncture + rehabilitation	Rehabilitation	Not described, 15 min	30 d	FMA
Mingxiang and Luo 2018 [32]	98	54.2 ± 2.3	54.2±	3.2 ± 1.3	3.1 ± 1.2	Not described	C	Not described	Acupuncture + rehabilitation	Rehabilitation	6 times/week, 30 min	2 M	MBI
Yaqing 2019 [45]	87	57.92 ± 4.83	58.25 ± 4.66	3.68 ± 0.72	3.51 ± 0.63	B	D	Not described	Acupuncture + rehabilitation	Rehabilitation	Once/day, 25–30 min	4 W	FMA, MBI
Fanglun 2015 [23]	60	64.47	65.23	29.77	30.63	Not described	Not described	G	Head acupuncture + rehabilitation	Rehabilitation	Once-twice/day	3 W	FMA
Shen 2012 [36]	100	59.2 ± 10.6	58.9 ± 8.1	Not described	Not described	Not described	C	Not described	Acupuncture + rehabilitation	Rehabilitation	Once/day, 30 min	3 W	FMA, MBI
Wang and Rehabilitation 2019 [38]	80	59.53 ± 13.52	58.50 ± 13.51	Not described	Not described	Not described	Not described	Not described	Acupuncture + rehabilitation	Rehabilitation	Not described, 30 min	3 M	FMA, MBI
Wang and Jiang 2006 [39]	146	55.7 ± 7.5	54.3 ± 7.2	Not described	Not described	B	D	Not described	Acupuncture + rehabilitation	Rehabilitation	6 times/week, 45 min	1 M	FMA, ND, MBI
Wang et al. 2017 [40]	130	60.5 ± 12.6	61.7 ± 10.9	3.3 ± 1.8	3.1 ± 1.5	Not described	C	K	Acupuncture + Western medicine	Western medicine	Once/day, 30 min	1 M	ND, MBI, FMA, QOL
Wong et al. 1999 [42]	118	60.4 ± 11.1	60.6 ± 10.8	Not described	Not described	Not described	Not described	Not described	Electroacupuncture + rehabilitation	Rehabilitation	5 times/week, 30 min	2 W	MBI
Xing Debing and Wang 2015 [43]	50	63.52 ± 13.23	61.48 ± 11.79	2.875 ± 2.501	2.798 ± 2.457	B	E	Not described	Electroacupuncture	Blank placebo	5–6 times/week, 30 min	2 W	MBI
Wei et al. 2012 [41]	90	64.22 ± 7.37	63.82 ± 7.54	11.22 ± 2.6	12.96 ± 15.92	Not described	C	Not described	Acupuncture + rehabilitation	Rehabilitation	6 times/week, 30 min	4 W	ND, MBI
Zhang et al. 2010 [35]	80	65.9 ± 11.1	69.2 ± 9.7	2.7 ± 1.47	3.4 ± 2.34	Not described	C	Not described	Acupuncture + rehabilitation	Rehabilitation	Once/day, 20 min	3 W	ND, MBI, MMSE
Haiyun 2009 [26]	72	63.7 ± 10.5	64.5 ± 10.6	7.0 ± 2.1	6.5 ± 2.4	Not described	C	Not described	Electroacupuncture + rehabilitation	Rehabilitation	Once/day, 30 min	4 W	FMA
Erjian 2018 [22]	50	74.2 ± 4.7	73.2 ± 3.9	Not described	Not described	Not described	Not described	Not described	Acupuncture + rehabilitation	Rehabilitation	Twice/day, 30 min	Not described	FMA, MBI

D: day, W: week, M: month, FMA: Fugl–Meyer Assessment scale, MBI: modified Barthel Index scale, MMSE: Mini-Mental State Examination scale, QOL: Quality Of Life Assessment scale, BBS: Berg Balance Scale, ND: Neurological Deficit Assessment scale, A: criteria for diagnosis and curative effect of diseases in traditional Chinese medicine, B: criteria for diagnosis and efficacy evaluation of stroke, C: diagnostic criteria formulated by the National Cerebrovascular Disease Conference, D: diagnostic points of various cerebrovascular diseases, E: Guidelines for Diagnosis and Treatment of Acute Stroke in China, F: neurological medicine, G: Stroke Classification Diagnosis and Treatment Guidelines of China in 2015, I: Guiding Principles for Clinical Research of New Chinese Medicines (Trial), J: Guidelines for Prevention and Treatment of Cerebrovascular Diseases in China, K: Brunnstrom rated in I-II.

## Data Availability

All data generated or used during the study are included in the submitted article.

## Supplementary Materials

Supplementary Materials The search strategy of WOS as an example.
