# Supplementary figures and images for: Acupuncture Therapy on Patients with Flaccid Hemiplegia after Stroke: A Systematic Review and Meta-Analysis
Source: Evid Based Complement Alternat Med. 2022 Jan 10;2022:2736703. doi: 10.1155/2022/2736703 (PMC8763511; doi:10.1155/2022/2736703)

Supplementary material: the search strategy of WOS as an example.


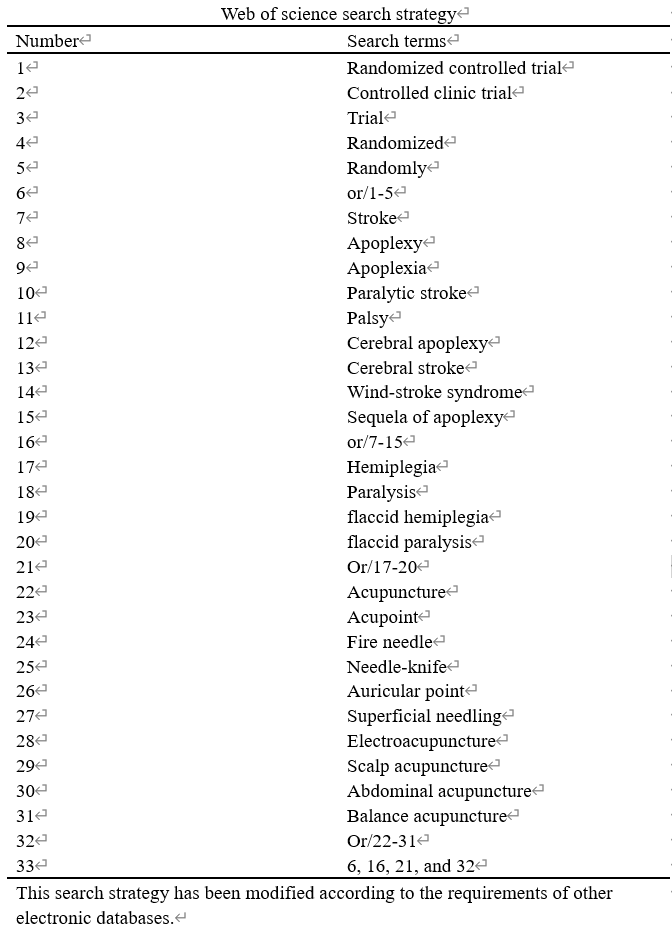

Supplement: Supplementary Materials — The search strategy of WOS as an example. [file 2736703.f1.docx]
